# Wild edible vegetables of ethnic communities of Mizoram (Northeast India): an ethnobotanical study in thrust of marketing potential

**DOI:** 10.1186/s13002-024-00680-1

**Published:** 2024-05-29

**Authors:** Rosie Lalmuanpuii, Betsy Zodinpuii, Beirachhitha Bohia, Zothanpuia, J. Lalbiaknunga, Prashant Kumar Singh

**Affiliations:** 1 Department of Botany, Mizoram University, Aizawl, Mizoram 796004 India; 2 Department of Zoology, Pachhunga University College, Mizoram, Aizawl 796005 India; 3 Department of Biotechnology/Life Sciences, Pachhunga University College, Mizoram University (A Central University), Mizoram, Aizawl 796001 India

**Keywords:** Wild edible vegetables, Direct matrix ranking, Informant consensus factor, Ethnobotany, Indigenous knowledge

## Abstract

**Background:**

Assessment of wild edible vegetables (WEVs) from the ethnobotanical approach is a significant key to understanding indigenous knowledge systems. The available literature has revealed a tremendous decline in knowledge of WEVs over the last few decades. The main purpose of this study was to document and analyse the traditional knowledge of WEVs among the two major ethnic groups of Mizoram regarding their use and the diversity associated with the importance of traditional medicines. Secondly, a market survey will be conducted to determine the status of available WEVs.

**Methods:**

This study conducted an ethnobotanical survey among 72 informants through semi-structured interviews and questionnaires besides direct field observations. The documented data were quantitatively analysed using various ethnobotanical indices, including Informant’s consensus factor (ICF), Fidelity level value (Fl), and Direct matrix ranking (DMR). A marketing survey was conducted in the Bara Bazar, Mizoram's most prominent local market. A total of 38 vendor informants were interviewed to observe and collect the price of commonly sold WEVs.

**Results:**

A total of 70 WEVs, distributed in 58 genera under 36 families, were documented and identified. Of these, 33 WEVs were of medicinal importance. Leafy vegetables were documented as the most frequently consumed parts (55.71%). The majority (44.29%) of the plants documented were consumed in fried form. The highest level of agreement among informants for food used categories was observed for plants combined with dry fish (ICF = 1). The Informant’s consensus factor (ICF) of disease categories ranges from 0.75 to 1, with the highest being reported for convulsion (ICF = 1), sleep inducer (ICF = 1), and antiseptic (ICF = 1). *Picria fel-terrae* was the most preferred plant for hypertension treatment (100% FL). Direct matrix ranking (DMR) indicated that *Dysoxylum excelsum* was highly utilized by the inhabitant for multipurpose species (DMR = 64). Jaccard similarity index (JI) between the two ethnic groups was revealed at 1.26. Forty-seven WEVs were found to be commercialized in the Bara Bazar market, Aizawl, with a price range from 0.1 to 2.4 USD. *Ensete superbum* was reported as near threatened per the IUCN Red List of Threatened Species.

**Conclusion:**

This work highlighted the importance and rich diversity of WEVs in Mizoram, which are presently used among different age groups for food and medicine. Informants have good knowledge of WEVs, which was shared to a great extent among the inhabitants; this legacy of traditional culture must be conserved. This study further suggests a priority setting for conserving multipurpose WEVs in human-inhabited sites, investigating the recorded species' nutritional properties and pharmacological activities.

## Introduction

The hilly state of Mizoram, one of the eight sisters of Northeast India, is a biodiversity hotspot in India [1]. It is popularly known as its home place for ‘Mizo’ ethnic communities diversified into seven major ethnic groups, either culturally or linguistically connected. About 60 per cent of the people are engaged in Slash and burn or jhum cultivation in Mizoram; at the same time, it is the primary source of income [2, 3]. However, shifting cultivation does not meet the basic requirements in periods of food shortage; at that time, wild edible vegetables (WEVs) played a significant role in compensating for the food crisis in Mizoram [4]. With globalization, the food crisis became more prominent, and forest resources, especially WEVs, became an important supplementary food source and medicine [5]. FAO also reported that over one billion people use WEVs as food sources [6]. In many developing countries, WEVs are important in filling food supply gaps during resource shortages and play an imperative role in maintaining and balancing the nutritional value and antioxidants in diets [7]. However, despite their importance as food, medicine, and many other purposes, traditional knowledge and practices of WEVs are being eroded due to urbanization and human activities across the globe, including India termo [8]. The impact of socio-cultural and environmental factors on wild food consumption [9, 10] has also been observed. These factors are also responsible for the negative impact on their biodiversity conservation [11]. Hence, ethnobotanical assessment is the key to understanding indigenous knowledge systems for future societies to overcome today's food crisis.

Nevertheless, the contribution of WEVs to the food system depends not only on the frequency of exploitation and the number of people consuming them but also on the agreement among the users [12]. Therefore, to get the reliable value of the particular plant species, quantitative ethnobotanical indices based on informant consensus were used to determine the agreement among the users [13]. There is also a strong correlation between the health and nutrition of WEVs, which is increasingly recognized [14]. Several studies have reported that WEVs are used for medicinal purposes [15, 16], and leaves are the main parts used to prepare medicine [17]. WEVs contain high nutraceuticals and are widely used to treat and prevent diseases like cancer, ulcers, inflammation, snakebite, asthma, diabetes, cardiovascular disease, gastritis, constipation, urinary problems, etc. [15, 17–20].

Notwithstanding the above, the WEVs also hold promise as a growing market segment, providing cash income and socio-economic development for the poor populace worldwide [21, 22]. Availability, market channels, and price information are crucial for accessing the importance of WEVs at local and national levels to complete the valuation of forest and forest products [23]. Moreover, WEVs have become a commercial crop in many developing countries zowith ever-increasing marketing potential [24]. As mentioned earlier, Mizoram, Northeast India, is inhabited by seven major ethnic communities, including Hmar, Paihte, Lai, Mara, Lushai, Chakma, and Bawm, dispersed in different districts, which more or less share the same culture and food habits [25]. In this study, two major ethnic groups, Hmar and Paihte, were selected to represent the Aizawl district and document the ethnobotanical knowledge of WEVs. Since the majority of Hmar and Paihte tribal groups reside in the Aizawl district, these two tribal groups, combining their environmental condition, cultural customs, and different linguistics, formed a unique traditional food culture and amassed rich traditional knowledge on the use of wild plant resources historically [25]. The indigenous Hmar and Paihte people in the study area are forest dwellers, solely dependent on farming, hunting, and shifting cultivation.

Moreover, due to poor transportation, lack of health care facilities, and insufficient supply of conventional crops, the residents rely on traditional medicines and WEVs. At the National level (India), the All-India Co-ordinated Research Project on Ethnobiology (AICRPE) documented valuable information that covered diverse tribal communities, such as Bagatas, Chenchus, Khonds, Konda Reddies, Koyas, and Sugalis. Recently, the health benefits of WEVs used by the rural people of Gondia district in Maharashtra [26], the di tribe of East Siang region of Arunachal Pradesh [27], the Jaunpur region of Uttarakhand [28] were investigated. In Mizoram, wild edible resources used by the Mizos [29] and the traditional medicinal plants that described their preparation and mode of use have been investigated qualitatively [30]. However, no comprehensive quantitative ethnobotanical investigation, documentation, or marketing potential of WEVs has been carried out for the Aizawl district, Mizoram. Given the information mentioned above, the present study was designed with the following objectives: (1) to conduct a comprehensive ethnobotanical investigation WEVs among the two ethnic groups of Mizoram, to fill the information gap in the documentation of WEVs and their uses, (2) evaluate the indigenous knowledge on ethnomedicinal uses of WEVs, to know the most valuable plants for primary health care, (3) analysed the gathered data by using various ethnobotanical indices, and compared with previous studies, and (4) marketing potential evaluation of the underutilized WEVs.

## Methodology

### Study area and site description

Mizoram (“Land of the Mizos”) situated in the high hills of the Northeast corner of India is a small state. It is bounded by Myanmar to the east and south, Bangladesh to the west and by the states of Tripura to the northwest, Assam to the north, and Manipur to the northeast. The state is endowed with a variety of landscapes, streams, and is rich in flora and fauna. The state has a rich cultural heritage which is reflected in its traditions and customs. More than three-fourths of the land area of Mizoram is forested. Thick evergreen forests containing valuable timber trees, wild food plants and medicinal plants. It covers an area of 21,081 Sq.km and has a population of 1,091,014 (2011 census). Topographically, the state has a pleasant climate, generally cool in summer and moderately cold in winter. The temperature varies from 20 °C to 29 °C in summer and from 11 to 21 °C during winter. The entire area is under the direct influence of the monsoon and the average rainfall is 254 cm per annum (Aizawl.nic.in). The residents of Mizoram consist almost entirely of Scheduled Tribes. These groups are called Mizo (“Highlander"). In the late nineteenth century, Christian missionaries began to work in the Mizo Hills area. Consequently, the majority of the population is Christian. Buddhists form the largest religious minority in Mizoram, followed by Hindu and Muslims. Agriculture such as jhum and terrace cultivation are the major economic activities of Mizoram [4]. Rice (*Oryza sativa* L.) is the staple food of the tribal groups of Mizoram, and it is engaged with vegetables, salads, and meats. Traditional Mizo foods are mainly organized in boiled, smoked, and fermented forms [4]. The famous cuisines include *bai* (a blend of vegetables and additional regional seasonings) and *tlak/mung* (boiled).

**Fig. 1:**
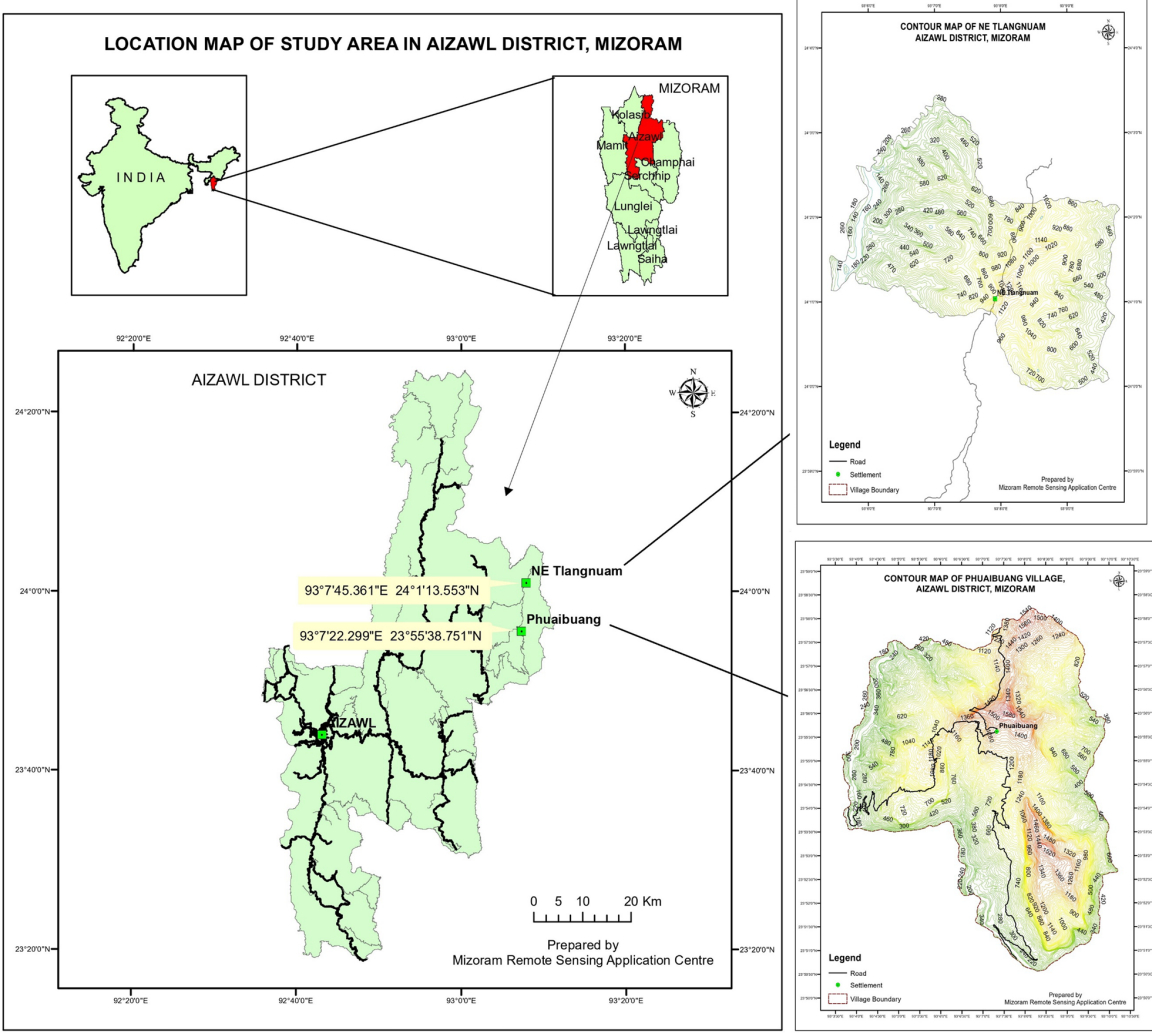
Geographical location of the study site: Phuaibuang and NE Tlangnuam, Aizawl district [Courtesy: Mizoram Remote Sensing Application Centre (MIRSAC)]

The study was confined to the Aizawl district of Mizoram, Northeast India (Fig. 1). Aizawl is the state capital of Mizoram. The total geographical area is 3576.31 km2 and accounts for 16.96% of the state's geographical location, comprising geographical features like agricultural plains and hilly terrains. The region is a mix forest type with tropical and subtropical semi-ever green forest. It has humid and moderate climatic conditions. [25]. There are three tribal residents in Aizawl district such as Hmar, Paihte, and Lusei. Within the Aizawl district, the two villages such as N.E Tlangnuam (93°7′45.361″E and 24°1′13.553″N,) and Phuaibuang (93°7′22.299″E and 23°55′38.751″N, altitude of 1392 m above sea level), were selected for ethnobotanical data collection. The prominent ethnic tribe of N.E Tlangnuam village is ‘Paihte’, and the common communication language is ‘Paihte tawng’ According to the 2011 census of Mizoram, it has a population of 658, with 318 males and 340 females residing in 97 households. It is 187 km away from the state capital Aizawl. The major ethnic tribe of Phuaibuang village is ‘Hmar.’ It constitutes 2134 populations (1087 male and 1047 female) with 398 households. Major communication languages are ‘Hmar’ and ‘Lusei’. It is 170 km away from the state capital, Aizawl. Phuaibuang is popularly known for its majestic peak just outskirts of the villages, named after an evergreen tree called ‘Hriangmual’. It is a home for a variety of flora and fauna like Deer, wild boar, Monkeys and many birds. There is also a Cave inside the Hriangmual forest known as ‘Fangfar Puk’, which is about 15/20 feet wide. There are Stalagmites inside this cave and water dripping from these stalagmites is collected by locals for medical use. It is believed that these waters have healing properties and are widely used for the treatment of various illnesses. The locals are strictly monitored for the protection and conservation of this beautiful Hriangmual Peak.

### Ethnobotanical data collection

The field survey was carried out from August 2015 to July 2016.

The Ethnobotanical data were documented through direct field observations, semi-structured interviews, and questionnaires [32]). Oral prior informed consent was acquired from the local informants before each interview following the International Society of Ethnobiology code of ethics http://ethnobiology.net/code-of-ethics/. The village head purposively introduced all the informants selected. The basic content of interviews followed the “5W + 1H” question pattern [33]. Data were collected using the major language, i.e. ‘Mizo,’ to get clear pictures of the knowledge about the plant species. Seventy-two local informants (41 female and 31 male) were interviewed with an age group of 25–70 years due to a lack of local knowledge regarding the use of WEVs among the younger generations. They are considered active groups, as suggested by the village head. The basic criterion for selecting these informants was their knowledge of utilizing WEVs, their nativity, and settlement duration in the study areas. All the chosen informants were of rural origin, implying that they were born in rural areas and raised in the same areas until adolescence. Moreover, the questionnaire focused on the local name, habit, part used, consumption mode, availability period, and ethnomedicinal uses of WEVs. Besides this, direct field observations were conducted with knowledgeable local people to collect the specimen, and photographs were taken for documentation.

### Identification of WEVs

The specimens (including flowers and fruits) collected were identified using a book of “Flora of Mizoram” [30], authenticated from BSI, Shillong, in 2016, and online databases such as World Flora Online http://www.worldfloraonline.org. The conservation status was recorded by referring to the data from the IUCN Red List version 2023-1 (https://www.iucnredlist.org/). Voucher specimens were deposited in the Department of Botany, Mizoram University, for future reference.

### Quantitative indices and data analyses

Data collected from informants on WEVs were analysed using various ethnobotanical indices, and all the data interpretations were calculated using MS Excel.

### Informant’s consensus factor (ICF)

ICF test was used to evaluate the homogeneity of knowledge about the species documented [34]. Before performing the analysis, the edible Phyto taxa were classified into 12 main categories and all the diseases were also classified broadly into twenty-two categories for the ailment category (Heinrich et al., 1998). CF was calculated as follows:

$${\text{ICF }} = {\text{Nur}} - {\text{Nt}}/{\text{Nur}} - {1}$$

Nur is the number of use reports from informants for a particular plant use category, and Nt is the number of species used for each category mentioned by all informants.

### Fidelity level (Fl)

To determine the most important species used by the local people in the study area, the Fidelity level (Fl) value was evaluated [35]. All the ailments were grouped into twenty-two classes (Table 2). Fl was calculated according to [36].

$${\text{Fl }}\left( \% \right) \, = \, \left( {NP/N} \right) \, \times { 1}00$$

Np is the number of informants that reported using plants to treat a particular disease. N is the number of informants who used the plants as medicine to treat any given disease.

### Direct matrix ranking (DMR)

DMR was evaluated to know the frequent multipurpose use of important WEVs and their utilization over dominance. Based on the virtual benefits attained from each selected thirteen edible species, five knowledgeable informants introduced by the village head were asked to assign values by giving order to each attribute among different uses such as medicinal, fodder, construction and fuel wood. Each selected Informant was invited to provide use values (5 = best, 4 = very good, 3 = good, 2 = less, 1 = least 0 = not used). The values given by the informants were summed up, and the rank was shown for each plant species [32].

### Jaccard index (JI)

Jaccard index (JI) was analysed to determine the similarity or dissimilarity of the WEVs used between the two ethnic communities.

$${\text{JI}} = \, C/A + B - C$$

where* C* is the number of species common to both ethnic groups, *A* is the number of species used only by one specific ethnic group, and *B* is the number of species used only by another ethnic group [37].

### Market surveys

A market survey was conducted in Bara bazaar, the biggest local market within the Aizawl district, to observe and collect the market price of commonly sold WEVs. During the investigations, a local market was regularly visited from January 2016 to December 2017, depending on the available season of the plants. Thirty-eight vendor informants who sell WEVs were interviewed (31 women and 7 men), and each vegetable's market price and season were collected verbally [13, 36].

## Results

### Demographic characteristics of informants

The study involved 72 informants from two ethnic groups, and their information on age and gender (Table 1**)**. The age distribution of informants from the two ethnic groups was segmented, and all the interviewees were between 25 and 70 years of age [Paihte people comprising 32 informants; male (15.28%) and females (25%) and Hmar people consist of 40 informants; male (23.61%) and females (31.94%)]. Female informants (56.94%) were more abundant than males (43.06) in the study area. Most informants were within the age range of 56–65 (41.67%).

**Table 1:** Demographic characteristics of the local informants in the study area

Category	Sub category	No. of informants	% of informants
Location	Phuaibuang village	40	55.56
	N.E Tlangnuam village	32	44.44
Gender	Male	31	43.06
	Female	41	56.94
Age (in years)	25–35	7	9.72
	36–45	12	16.67
	46–55	15	20.83
	56–65	30	41.67
	66–70	8	11.11
Occupation	Farmer	35	48.61
	Animal husbandry	11	15.28
	Unemployed	7	9.72
	Teacher	9	12.5
	Others	10	13.89

### Plant species, use reports, and life form distributions

**Fig. 2:**
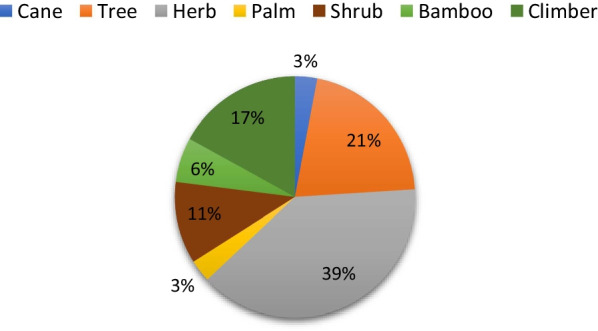
Life-form distributions of documented WEVs

The study recorded 70 WEVs belonging to 36 families and 58 genera as utilized by the two ethnic communities of Aizawl district, Mizoram **(**Table 2**)**. Among the species documented, Cucurbitaceae and Fabaceae represent the highest number of species (5 species each). The highest number of genera comprise *Solanum* (4 species), followed by *Acmella, Amaranthus, Aralia, Calamus, Caryota, Dendrocalamus, Dioscorea, Marsdenia, Momordica,* and *Musa* (2 species each), and the remaining genera constitute one species each**.** The informants refer 1416 UR of 70 taxa, 489 UR (39.56%) of them corresponding to the food use categories, 747 UR (60.44%) to the medicinal uses, and 180 UR (12.71%) to the other uses. The mean number of food use and medicinal taxa cited by the informants were 9.58 and 3.5, respectively. Seventy species of WEVs were dominated by herbs (39%), followed by trees (21%), climbers (17%), shrubs (11%), and bamboo (6%), and a minimum was recorded for palms and canes (3% each) **(**Fig. 2**).**

**Table 2:** List of WEVs documented during field survey with local name, family, life form, available season, part use(s), mode of consumption, IUCN GCS* Ethnic groups: H- Hmar, P- Paihte

Sl/no	Botanical name/ voucher codes	Local name	Family	Life-Forms	Available season	Part use(s)	Mode of consumption	Other local uses	IUCN GCS	References
1	*Acmella* *oleracea* (L.) R.K.Jansen /MZUBOT0201	P; Ansalai H; Ansapui	Asteraceae	Herb	Feb–Nov	Leaves/ shoots	Salad/fried/ Boiled	Fodder, medicine	LC	Kar et al. [29]
2	*Acmella* *paniculata* (Wall. ex DC.) R.K.Jansen/MZUBOT0202	P; Ansa malngat	Asteraceae	Herb	Feb–Nov	Leaves/ shoots	Boiled/ Fried/ Salad	Medicine, fodder	LC	Konsam et al. [31] Kar et al. [29]
3	*Aganope thyrsiflora* (Benth.) Polhill. /MZUBOT0203	H, P; Hulhu	Fabaceae	Climber	Feb–Apr	Leaves/Shoots	Boiled/ fried	Fuelwood	NE	Kar et al. [32]
4	*Alocasia* fornicata (Kunth) Schott/MZUBOT0204	H, P; Baibing	Araceae	Herb	Jul–Sep	Flower bud	Boiled/ Fried	Fodder	LC	Kar et al. [32]
5	*Alternanthera sessilis* (L.) DC. /MZUBOT0205	H;An-ngharil P; Nghagilkhate	Amaranthaceae	Herb	Mar–Sep	Leaves	Fried		LC	Kar et al. [32], Chauhan et al.[33]
6	*Amaranthus spinosus* L./ MZUBOT0206	P; Lenling nei	Amaranthaceae	Herb	Apr–Sep	Leaves/Shoots	Combined with other vegetables	Fodder	NE	Kar et al. [32]
7	*Amaranthus viridis* L./ MZUBOTO207	H; Lenhling hling neilo P; Lenling neilo	Amaranthaceae	Herb	Apr–Sep	Leaves /Shoots	Combined with other vegetables	Fodder, medicine	NE	Kar et al.[34], Singh et al. [35]
8	*Amomum dealbatum* Roxb./MZUBOT0208	H; Aihri (Aidu) P;Aigechil	Zingiberaceae	Herb	Jan–Apr	Inflorescence	Boiled/Fried	Medicine, crafting	DD	Kar et al. [29]
9	*Amorphophallus napalensis* (Wall.) Bogner &Mayo/ MZUBOT0209	H, P; Telhawngpa	Araceae	Herb	Aug–Dec	Tuber	Boiled/Fried	Fodder	NE	Kar et al. [29]
10	*Antidesma bunius*( L.) Spreng./ MZUBOT0210	P; Tuaitit H; Mang Tuaitit	Euphorbiaceae	Tree	Mar–Aug	Leaves	combined with meat	Fuelwood, fodder, construction, medicine	LC	Kar et al. [29]
11	*Aralia foliolosa* Seem. ex C.B.Clarke/ MZUBOT0211	H;Hlingthufir P;Lingdawng	Araliaceae	Shrub	Mar–Aug	Leaves /Shoots	Boiled/Fried	Fuelwood	LC	Kar et al. [29]
12	*Aralia dasyphylla* Miq./MZUBOT0212	H; Hlingthufir suak P; Lingdawng suag	Araliaceae	Shrub	Mar–Aug	Leaves /Shoots	Boiled/Fried	Fuelwood	LC	–
13	*Asparagus officinalis* L./MZUBOT0213	H; Thingribuk	Asparagaceae	Climber	Apr–Sep	Shoot	Fried	Medicine	LC	Konsam et al. [31], Thakur et al. [36], Kar et al. [29]
14	*Azadirachta indica* A. Juss/ MZUBOT0214	H, P; Neem	Meliaceae	Tree	Jan–Oct	Leaves	Fried/ Salad	Fuelwood, construction, medicine	LC	Bhagat et al. [37]
15	*Bambusa tulda* Roxb/ MZUBOT0215	H: Rawthing P: Rawting	Poaceae	Bamboo	May–Oct	Tender shoot	Combined with fermented pork	Fuelwood, crafting	NE	–
16	*Blumea myriocephala* (DC.)H. Rob. / MZUBOT0216	H, P; Buardap	Asteraceae	Herb	Aug–Nov	Leaves	Boiled	Fodder	NE	–
17	*Brassaiopsis* *hainla* (Buch. -Ham.) Seem. /MZUBOT0217	H; Antumbu P; Antumbu	Araliaceae	Tree	Apr–Sep	Leaves/Shoot	Boiled	Fencing	NE	Meitei et al. [38]
18	*Calamus erectus* Roxb. / MZUBOT0218	H; Hruizik P; Chingzik	Arecaceae	Cane	whole year	Tender shoot	Boiled	Crafting	LC	Kar et al. [29]
19	*Calamus tenuis* Roxb.* /* MZUBOT0219	H, P; Thilthek	Arecaceae	Cane	whole year	Tender shoot	Boiled	Crafting, medicine	LC	Kar et al. [29]
20	*Caryota mitis* Lour. / MZUBOT0220	H; Meihle P; Meile	Arecaceae	Palm	whole year	Shoots	Boiled	Ornamental	LC	Kar et al. [29]
21	*Caryota urens* L./MZUBOT0221	H, P; Tum	Arecaceae	Palm	whole year	Shoots	Boiled	Ornamental	LC	Kar et al. [29], Meitei et al. [38]
22	*Centella asiatica* L./ MZUBOT0222	H; Lambak P; Lambak	Umbelliferae	Herb	Apr–Oct	Whole plants	Raw	Fodder, medicine	LC	Konsam et al. [31], Kar et al. [29]
23	*Chenopodium album* (L.) Urb./ MZUBOT0223	H, P; Kawlbuh	Amaranthaceae	Herb	Mar–May	Leaves /Shoot	Combined with rice	Medicine	NE	Konsam et al. [31], Thakur et al. [36], Kar et al. [29]
24	*Clerodendrum* *bracteatum* Wall./ MZUBOT0224	H, P; Phuihnam/ Anphui	Lamiaceae	Tree	Jan–Oct	Leaves	Combined with meat/ other vegetables	Medicine, fuelwood, fencing	NE	Konsam et al. [31], Kar et al. [29]
25	*Cordia dichotoma* G. Forster /MZUBOT0225	P; Muk	Boraginacea	Tree	April–Oct	Leaves	combined with meat	Timber, fuelwood, medicine	LC	Kar et al. [29], Chauhan et al. [33], Bhatia et al. [39]
26	*Crotalaria tetragona* Roxb. Ex Andr. / MZUBOT0226	H, P; Tumthang	Fabaceae	Shrub	Sep–Dec	Flowers	Combined with meat		NE	Kar et al. [29]
27	*Dendrocalamus hamiltonii* Nees & Arn. ex Munro/MZUBOT0227	H; Phulrua P; pul lua	Poaceae	Bamboo	May–Oct	Tender shoot	Combined with fermented pork	Construction, fuelwood, fodder	NE	Kar et al. [29]
28	*Dendrocalamus longispathus* (Kurz)/MZUBOT0228	H; Rawnal P;Rawnal	Poaceae	Bamboo	May–Oct	Tender shoot	Combined with fermented pork	Construction	NE	Deb et al.[40]
29	*Dioscorea alata* L./MZUBOT0229	H, P; Ram bahra	Dioscoreaceae	Climber	Whole year	Tuber	Boiled	Fodder	NE	Meitei et al. [38]
30	*Dioscorea bulbifera* L/ MZUBOT0230	H;Bachhim	Dioscoreaceae	Climber	Aug–Nov	Tuber	Boiled	Medicine	NE	Kar et al. [29]
31	*Diplazium esculentum* / (Rets.) Sw. MZUBOT0231	H, P; Chakawk	Athyriaceae	Herb	Mar–Nov	Leaves	fried/boiled/ Salad		LC	Konsam et al. [31], Thakur et al. [36], Kar et al. [29]
32	*Dysoxylum excelsum* Blume/ MZUBOT0232	H; Thingthupui P; Singthupi	Meliaceae	Tree	Apr–Sep	Leaves/shoots	Boiled/ Fried	Medicine, fodder, construction, fuelwood	NE	Konsam et al. [31] Kar et al. [29]
33	*Elatostema* *rupestre* (Buch. -Ham. ex D.Don) Wedd /MZUBOT0233	H;Mangmanmim	Urticaceae	Herb	Whole year	Leaves	Combined with rice		NE	–
34	*Ensete* *superbum* (Roxb.) Cheesman /MZUBOT0234	H; Saisua/saisu P; Saisuang	Musaceae	Herb	Whole year	Shoots	Combined with fermented pork	Medicine, fodder	NT	Kar et al. [29]
35	*Eryngium foetidum* L/*.* MZUBOT0235	H, P; Bachikhawm	Apiaceae	Herb	Mar–Nov	Whole plant	Raw	Medicine	LC	Konsam et al. [31]
36	*Eurya acuminata.*DC/ MZUBOT0236	H, P; Sihzo/ Sihneh	Pentaphylacaceae	Tree	whole year	Leaves	Combined with rice Dried form /Combined with meat	Construction, fuelwood, fencing	NE	Konsam et al.,2016, Kar et al. [29]
37	*Fagopyrum* *tataricum* (L.) Gaertn. / MZUBOT0237	H, P; Anbawng	Polygonaceae	Herb	Mar–Sep	Leaves	Combined with fermented pork	Medicine, fodder	NE	Konsam et al. [31], Thakur et al. [36], Kar et al. [29]
38	*Ficus auriculata* Lour. /MZUBOT0238	H; Theibal	Moraceae	Tree	Whole year	Leaves	Cook with dry fishes	Construction, fuelwood	LC	Thakur et al. [36], Kar et al. [29]
39	*Glinus* *oppositifolius* Aug. DC. / MZUBOT0239	H; Bakhate	Molluginaceae	Herb	Apr–Nov	Leaves	Fried	Medicine	LC	Kar et al. [29]
40	*Gnetum gnemon* L*/* MZUBOT0240	H; Pelh	Gnetaceae	Herb	Jun–Sep	Leaves	Fried	Fuelwood, fodder	LC	Kar et al. [29]
41	*Gynura* *cusimbua* S. Moore/ MZUBOT0241	H; Tlangnal P; Tangnal	Asteraceae	Herb	Mar–July	Leaves/ shoots	Fried	Fodder	NE	Kar et al. [29]
42	*Houttuynia cordata* Thunb. / MZUBOT0242	H, P; Uithinthang	Saururaceae	Herb	Feb–Aug	Leaves	Salad	Medicine	NE	Konsam et al. [31]
43	*Lepionurus sylvestris* Blume/ MZUBOT0243	H; Anpangthuam P: Anpangthuam	Opiliaceae	Shrub	Whole year	Tender leaves	Raw/ cooked with fermented pork	Fuelwood, construction, medicine	NE	Konsam et al. [31]
44	*Luffa* *acutangula* Roxb./ MZUBOT0244	H, P; Awmpawng	Cucurbitaceae	Climber	Apr–Aug	Fruits	Fried		NE	Kar et al. [29], Bhagat et al. [37]
45	*Marsdenia formosana* Masam/MZUBOT0245	H; Phai ankhate P; Ankhaneu	Apocyanaceae	Climber	whole year	Leaves / Shoots	Cook with fermented pork	Medicine	NE	Kar et al. [29]
46	*Marsdenia maculata* Hook. /MZUBOT0246	H; Ankhapui P; Ankhapi	Apocyanaceae	Climber	whole year	Leaves / Shoot	Cook with fermented pork	Medicine	NE	Kar et al. [29]
47	*Meloccana baccifera (*Roxb.) Kurz /MZUBOT0247	H, P: Mautak	Poaceae	Bamboo	Apr–Sep	Shoots	Pickle/ Fried/ Combined with fermented pork/ meat/ Boiled/ dried form	Fodder, fuelwood, construction	NE	Kar et al. [29], Meitei et al. [38]
48	*Momordica charantia* L./ MZUBOT0248	H; Kharek/ Changkhate P; Tangkhamal neu	Cucurbitaceae	Climber	Apr–Oct	Fruits/ Leaves	Fried	Medicine	NE	Kar et al. [29]
49	*Momordica* *dioica* Roxb. ex Willd./ MZUBOT0249	H, P; Maitamtawk	Cucurbitaceae	Climber	May–Jun	Fruits	Fried/boiled		NE	Konsam et al. [31], Bhagat et al. [37]
50	*Morus indica* L./ MZUBOT0250	P; Thing theihmi	Moraceae	Tree	Whole year	Leaves	combined with meat	Fodder, fuelwood, construction	LC	Kar et al. [29]
51	*Musa balbisiana* Colla/ MZUBOT0251	H; Tumbu P; Nahtangum	Musaceae	Herb	whole year	Inflorescence	Boiled/ Pickle/ Combined with fermented soya bean	Fodder, construction, medicine	LC	Kar et al. [29]
52	*Musa paradisiaca* L./ MZUBOT0252	H P; Lairawk	Musaceae	Herb	whole year	Inflorescence	Boiled/ Pickle/ Combined with fermented soya bean	Fodder, medicineconstruction	LC	–
53	*Oroxylum indicum* (L.) Kurz /MZUBOT0253	H, P; Pualchangkawk/ Archangkawm	Bignoniaceae	Tree	Aug–Nov	Fruits	Salad	Fuelwood, construction, medicine	NE	Kar et al. [29]
54	*Parkia* *timoriana* (DC.) Merr/. MZUBOT0254	H, P; Zawngtah	Fabaceae	Tree	Oct–Apr	Fruits	Raw/ fried/ mixed with fermented pork	Medicine, fuelwood, construction	NE	Kar et al. [29]
55	*Picria fel-terrae *Lour*./* MZUBOT0255	H, P; Tlungkha/ khatual	Linderniaceae	Herb	May–Dec	leaves / Shoot	Combined with meat, Dried form	Medicine	NE	–
56	*Plantago major* L./ MZUBOT0256	H; Vawkna-an P; Vawkbilte	Plantaginaceae	Herb	Mar–Sep	Leaves	fried	Medicine	NE	Konsam et al. [31], Kar et al. [29]
57	*Portulaca oleracea* L./ MZUBOT0257	H; Bawk ek an	Portulacaceae	Herb	Mar–Nov	Whole plant	Fried	Medicine, fodder	NE	Konsam et al. [31], Thakur et al. [36], Singh et al. [41]
58	*Rhynchotechum ellipticum* (Wall. ex D.Dietr.) A.DC /MZUBOT0258	H; Tiarrep P;Chiaklep	Gesneriaceae	Shrub	Apr–Aug	Leaves	Combined with fermented soybeans or pork	Medicine, fodder	NE	Kar et al. [29]
59	*Senegalia pennata* (L.) Maslin/ MZUBOT0259	H; Khanghu P; Khanghmuk	Fabaceae	Tree	Mar–May	Leaves/Shoot	Boiled/fried	Fuelwood, fencing, medicine	LC	Kar et al. [29]
60	*Senna occidentalis* L./ MZUBOT0260	H; Rengan P; Lengan	Fabaceae	Shrub	May–Jul	Leaves	Combined with rice		NE	Kar et al. [29]
61	*Solanum americanum* Mill./ MZUBOT0261	H; Mit-thut/Anhling P; An zo	Solanaceae	Herb	Feb–Oct	Leaves / Shoot	Fried/ Boiled	Fodder, medicine	NE	Konsam et al. [31], Kar et al. [29]
62	*Solanum torvum* Sw./ MZUBOT0262	H; Tawkpui P; Samphawk pi	Solanaceae	Shrub	May–Jul	Fruits	Fried/Raw	Fuelwood, medicine	LC	Konsam et al. [31]
63	*Solanum* *anguivi* Lam./ MZUBOT0263	H; Tawkte P; Samphawk neu	Solanaceae	Shrub	Apr–Jul	Fruits	Boiled with other vegetables	Medicine	LC	Kar et al. [29]
64	*Solanum* *lycopersicum* L. Dunal /MZUBOT0264	H, P; Tomato te	Solanaceae	Climber	Feb–Dec	Fruits	raw/salad		NE	Kar et al. [29]
65	*Solena heterophylla* Lour. / MZUBOT0265	H;Uiluvun	Cucurbitaceae	Climber	Apr–Jun	Leaves/Shoot	Cook with fermented pork		NE	–
66	*Thladiantha* *cordifolia* Cogn /MZUBOT0266	H; kangmang P; Mang kang	Cucurbitaceae	Climber	Sept–Apr	Leaves	Combined with fermented pork		NE	Kar et al. [29]
67	*Trevesia palmata* (Roxb. ex Lindl.) Vis./ MZUBOT0267	H; Kawhtebel P; Uilusing	Araliacea	Tree	Feb–April	Flower bud	Boiled/ Fried	Construction	LC	Kar et al. [29]
68	*Sauromatum horsfieldii *Miq./ MZUBOT0268	H, P; Telhawngnu	Araceae	Herb	Aug–Dec	Bulb	Boiled/ Fried	Fodder	NE	Kar et al. [29]
69	*Wendlandia budleioides* Wall.ex Wight&Arn / MZUBOT0269	H; Ba-tling P; Bating	Rubiaceae	Tree	Feb–April	Flower bud	Boiled/ Fried	Fuelwood, fodder, construction	NE	Konsam et al. [31] Kar et al. [29]
70	*Zanthoxylum rhetsa* (Roxb).Dc/MZUBOT0270	H, P: Chingit	Rutaceae	Tree	Aug–Dec	Leaves /shoot	Combined with other vegetables or fermented soya bean/ fried		LC	Konsam et al. [31], Doni and Gajurel. [42]

### Plant parts consumed

**Fig. 3:**
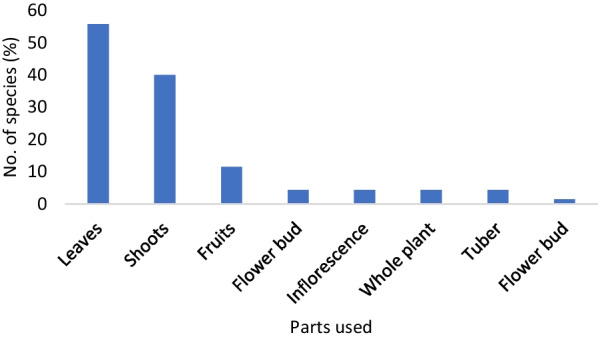
Frequency of plant parts used as vegetables among the two ethnic communities

The study revealed that the inhabitants consumed different plant parts in the study area. Out of 70 species, the leafy vegetable (55.71%) was the most frequently consumed plant parts (39 species), followed by shoot vegetables (28 species, 40%), fruits (8 species, 11.43%), flower bud, inflorescence, whole plants and tuber (3 species each, 4.29%). There was one species (1.43%) where the flower was taken as a vegetable **(**Fig. 3**)**.

### Modes of consumption

**Fig. 4:**
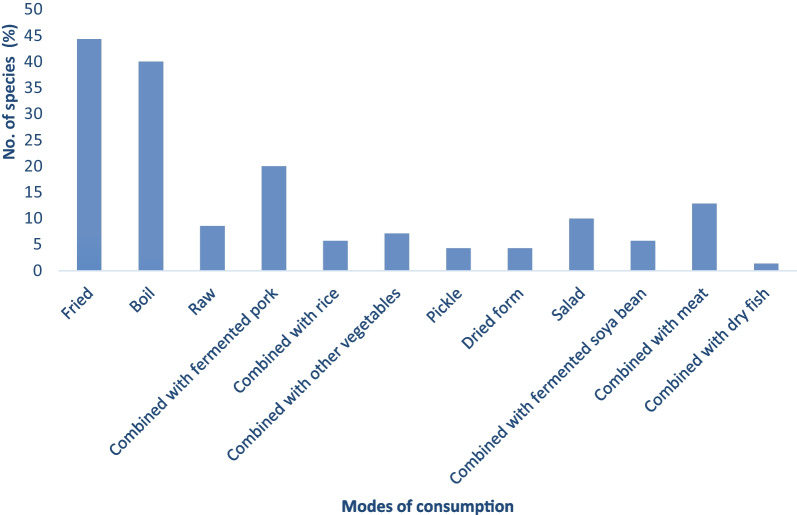
Different modes of consumption of WEVs

Regarding the modes of consumption of WEVs, 31 plants (44.29%) were consumed in fried form, 28 plants (40%) in boiled form, 14 plants (20%) in combination with fermented pork, 9 plants (12.86%) in combination with meat, 7 plants(10%) as salad preparations, 6 plants (8.57%) in raw, 5 plants (7.14%) in combination with other vegetables, 4 plants (5.71%) each were cooked in combination with rice and with fermented soya bean, 3 plants (4.29%) as pickle and after sun-dried, respectively, while 1 plant (1.43%) was consumed by combining with dried fish **(**Table 2**; **Fig. 4**)**.

### Seasonal availability of WEVs

**Fig. 5:**
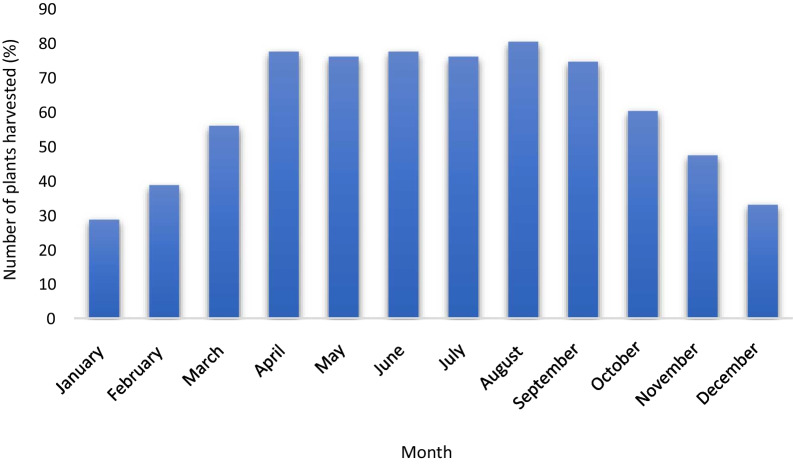
Seasonal availability of WEVs in the study site

WEVs were available throughout the year **(**Table 1**; **Fig. 5**)**. Most of the WEVs investigated were harvested by the local people from April to September, mainly during the rainy season. The peak availability was observed in August when 80% of the plant species were available for harvest. Minimum WEVs were harvested during the dry period, January (28.57%) and December (32.86%), where the amount of rainfall decreased during this period.

### Informant consensus factor (ICF) of food used category

**Fig. 6:**
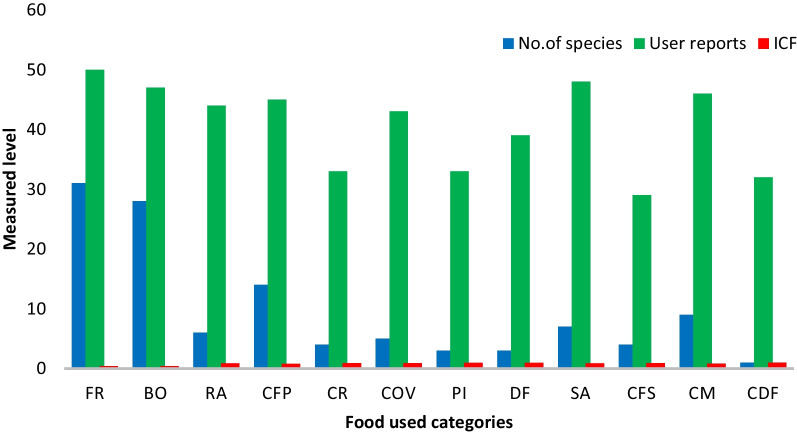
Informant consensus factor (ICF) of food used category with user reports (UR) and number of species from the study area (FR—fried form, BO—boil, RA—raw, CFP—combined with fermented pork, CR—combined with rice, COV—combined with other vegetables, PI—pickle, DF—dried form, SA—salads, CFS—combined with fermented soya bean, CM—combined with meat, CDF—combined with dried fish)

To understand the level of agreement among informants of the two ethnic communities, the Informant’s consensus factor (ICF) was evaluated. In this study, ICF for the food used categories ranges from 0.39 to 1.00. The category with the highest ICF was found in combination with dry fish (1.00), having 32 UR for 1 plant species, followed by dried form (0.95), having 39 UR for 3 species, and so on **(**Fig. 6**)**. The least agreement among informants was observed for plants used in fried form (0.39), with 50 UR for 31 species. The high ICF value signified that the informants used fewer taxa to make food.

### Ethnomedicinal uses of WEVs

**Fig. 7:**
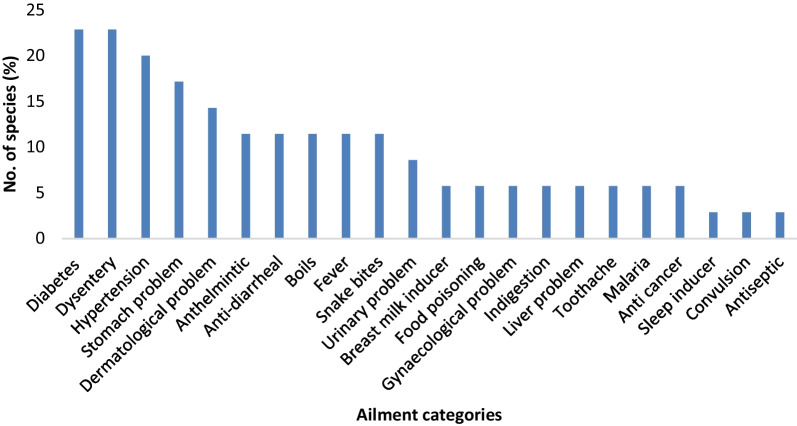
Distribution frequency of the number of food medicines reported by the informants based on the ailments they can be treated

Investigation and documentation of WEVs showed that the local inhabitants used certain ethnomedicinal plants as food **(**Table 3**)**. The local informants mentioned 35 WEVs as medicine to treat several diseases. Eight plant species (22.86%) each were used to treat diabetes and dysentery, 7 species (20%) for hypertension, 6 species (17.14%) for stomach problems, 5 species (14.29%) for dermatological issues, 4 species (11.43%) each for anthelmintic, anti-diarrheal, boils, fever, and snake bites, 3 species (8.57) to treat the urinary problem, 2 species each (5.71%) were used as breast milk inducer, food poisoning, gynaecological problem, indigestion, liver problem, toothache, anti-cancer and malaria, 1 species each (2.86%) as an antiseptic, sleep inducer and convulsion **(**Fig. 7**)**.

**Table 3:** WEVs used as traditional medicine reported by informants from the study site

Sl/No	Diseases category	Plants used
1	Anthelmintic	*Acmella paniculata, Solanum americanum, Zanthoxylum rhetsa, Acmella oleraceae*
2	Anti- Cancer	*Rhynchotechum ellipticum, Dioscorea bulbifera*
3	Anti-diabetic	*Musa balbisiana, Momordica charantia, Centella asiatica, Picria fel-terrae, Dioscorea bulbifera, Oroxylum indicum, Plantago major, Lepionurus sylvestris*
4	Anti-diarrheal	*Musa balbisiana, Dysoxylum excelsum, Parkia timoriana Fagopyrum tataricum,*
5	Antiseptic	*Solanum anguivi*
6	Boils	*Solanum anguivi, plantago major, Solanum torvum, Solanum americanum,*
7	Breast milk inducer	*Momordica charantia, Glinus oppositifolius,*
8	Convulsion	*Ensete superbum*
9	Dermatological problem	*Picria-fel- terrie, Solanum anguivi, Solanum torvum, Azadirachta indica, portulaca oleracea,*
10	Dysentery	*Musa paradisiaca, Oroxylum indicum, Aganope thyrsifolia, Dysoxylum excelsum, Calamus tenuis, Asparagus officinalis, Parkia timoriana, Eryngium foetidum*
11	Fever	*Ensete superbum, Picria fel-terrae, Solanum anguivi, Plantago major*
12	Food poisoning	*Senegalia pennata, Dysoxylum excelsum*
13	Gynae problem	*Cordia dichotoma, Fagopyrum tataricum*
14	Hypertension	*Momordica charantia, Clerodendrum bracteatum, Centella asiatica, Marsdenia macrophylla, Marsdenia formosana. Senna occidentalis, Picria fel-terrae*
15	Indigestion	*Fagopyrum tataricum, Parkia timoriana*
16	Liver problem	*Ensete superbum, Glinus oppositifolius*
17	Malaria	*Plantago major, Eryngium foetidum*
18	Sleep inducer	*Amomum dealbatum*
19	Snake bites	*Musa balbisiana, Amaranthus viridis, Ensete superbum, Antidesma bunius*
20	Stomach problem	*Oroxylum indicum, Centella asiatica, Aganope thyrsiflora, Fagopyrum tataricum, Eryngium foetidum, Azadirachta indica*
21	Toothache	*Acmella oleraceae, Solanum americanum*
22	Urinary problem	*Solanum americanum, Senna occidentalis, Portulaca oleracea*

### Informant’s consensus factor (ICF) of disease categories:

**Fig. 8:**
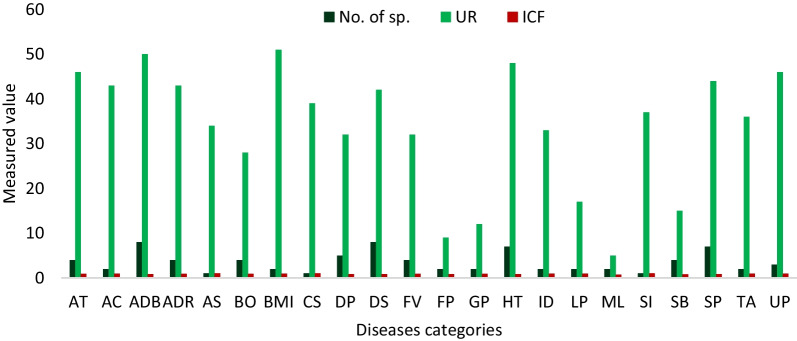
Informant’s consensus factor (ICF) of various diseases indications; UR—User reports, AT—Anthelmintic, AC—Anti-cancer, ADB—Anti-diabetic, ADR—Anti-diarrheal, AS—Antiseptic, BO—Boils, BMI—Breast milk inducer, CS—Convulsion, DP—Dermatological problem, DS—Dysentery, FV—Fever, FP—Food poisoning, GP—Gynae problem, HT—Hypertension, ID—Indigestion, LP—Liver problem, ML—Malaria, SI—Sleep inducer, SB—Snake bites, SP—Stomach problem, TA—Toothache, UP—Urinary problem

The ICF value of WEVs used as ethnomedicine was calculated after being grouped into twenty-two categories based on the user report (UR)**.** The results showed that F_ic_ ranges from 0.75 to 1, with the highest report for convulsion (ICF = 1; 1 species; 39 UR), sleep inducer (ICF = 1;1 species; 37 UR), and antiseptic (ICF = 1; 1 species; 34 UR). The least agreement among informants was evaluated for the plants used to treat snake bites (ICF = 0.79; 4 species; UR = 15) and malaria (ICF = 0.75; 2 species; 5 UR) **(**Fig. 8**)**.

### Fidelity level value (%) of commonly reported ethnomedicinal plants

Fidelity level (Fl) values help identify the most preferred and important plant species for treating a disease or ailment. The Fl value was calculated for the 35 most common ethnomedicinal plants reported by the informants within the study area*.* The study reports the Fl values varying from 30.77 to 100% in all the disease categories. The highest Fl values were obtained for *Picria fel- terrae* (100%), implying that it was the most preferred ethnomedicinal plant by the informants to treat hypertension. This was followed by *Solanum anguivi* (Fl = 97%) and *Picria fel-terrae* (95%), which were recommended for treating diabetes. On the contrary, the least Fl value was recorded for *Senegalia pennata* (30.77%) to treat food poisoning **(**Table 4**)**.

**Table 4:** Fidelity level (Fl) values of medicinal plants commonly reported against various diseases/ailment categories

Ethnomedicinal plants	Diseases categories	Np	N	Fl (%)
*Picria fel-terrae*	Anti-diabetic	19	20	95
*Momordica charantia*		28	30	93
*Lepionurus sylvestris*		12	15	80
*Musa balbisiana*	Diarrhoea	16	24	66.67
*Dysoxylum excelsum*		18	19	94.74
*Musa balbisiana*	Dysentery	9	15	60
*Eryngium foetidum*		12	27	44.44
*Dysoxylum excelsum*		21	23	91.30
*Musa balbisiana*	Snake bites	21	32	65.63
*Ensete superbum*	Fever	18	35	51.43
*Ensete superbum*	Convulsion	39	47	82.98
*Oroxylum indicum*	Stomach problem	16	20	80
*Fagopyrum tataricum*		13	16	81.25
*Clerodendrum bracteatum*	Hypertension	20	22	90.91
*Centella asiatica*		15	19	78.95
*Picria fel-terrae*		14	14	100
*Momordica charantia*	Breast milk Inducer	35	61	57.38
*Glinus oppositifolius*		39	45	86.67
*Solanum anguivi*	Antiseptic	34	35	97.14
*Solanum anguivi*	Boils	21	25	84
*Solanum americanum*		19	23	82.61
*Portulaca oleracea*	Urinary problem	20	30	66.67
*Solanum americanum*		26	35	74.29
*Rhynchotechum ellipticum*	Anti-cancer	12	17	70.59
*Dioscorea bulbifera*		31	45	68.89
*Acmella oleracea*	Toothache	28	36	77.78
*Acmella oleracea*	Anthelmintic	44	50	88
*Fagopyrum tataricum*	Indigestion	23	36	63.89
*Parkia timoriana*		10	18	55.56
*Fagopyrum tataricum*	Gynaecological problem	6	15	40
*Senegalia pennata*	Food poisoning	4	13	30.77
*Eryngium foetidum*	Malaria	4	11	36.36
*Solanum anguivi*	Dermatological problem	9	14	64.29
*Azadirachta indica*		11	25	44
*Amomum dealbatum*	Sleep inducer	37	50	74
*Ensete superbum*	Liver problem	10	19	52.63

*FL* Fidelity level, *Np*= Number of informants that reported using plants to treat a particular disease, *N* Number of informants who used the plants as a medicine to treat any given disease

### Direct matrix ranking (DMR)

In this study, 13 multipurpose species of WEVs were selected, and 4 used categories were listed for 5 informants to assign the multipurpose use of the species. Accordingly, *Dysoxylum excelsum* was highly utilized by the inhabitant for multipurpose and ranked first (DMR = 64); *Antidesma bunius* (60) ranked second; *wendlandia budleioides* (56) ranked third. The lowest rank was observed for *Rhynchotechum ellipticum* (30) **(**Table 5**)**.

**Table 5:** Average DMR score of five informants for most commonly used WEVs

Species	Use categories					
	Fuelwood	Fodder	Construction	Medicine	Total	Rank
*A. oleraceae*	0	23	0	21	44	7th
*A. bunius*	21	15	12	18	60	2nd
*A. indica*	22	0	10	23	50	5th
*B. tulda*	4	7	20	0	31	11th
*D. hamiltonii*	8	7	19	0	34	10th
*D. excelsum*	25	0	15	25	64	1st
*E. acuminata*	23	15	6	0	41	9th
*F. tataricum*	0	23	0	21	44	7th
*M. baccifera*	7	12	23	0	43	8th
*M. balbisiana*	0	25	3	21	51	4th
*R. ellipticum*	0	9	0	21	30	12th
*S. americanum*	0	20	0	25	45	6th
*W. budleioides*	4	18	14	0	56	3rd

Based on use criteria (5=best; 4=very good; 3=good; 2=less used; 1=least used; 0=no value)

**Fig. 9:**
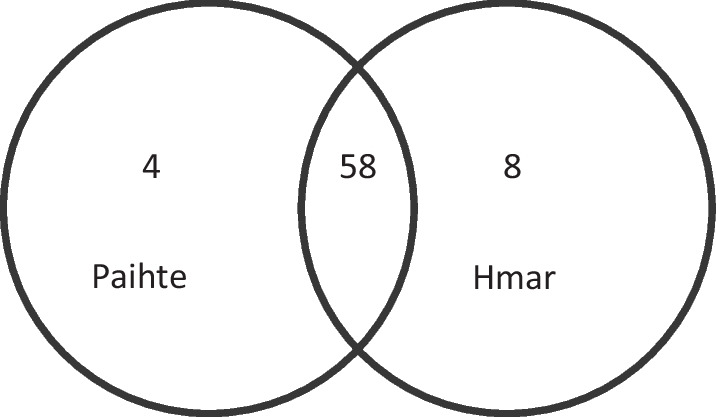
Venn diagram showing Jaccard similarity index (JI) and overlap of reported WEVs among the studied ethnic groups

Jaccard similarity index (JI):

The result of the similarity index for WEVs used between the two ethnic groups was revealed at 1.26. Of all the 70 species cited by the informants, 58 WEVs were shared among both ethnic groups, 4 species are only used by the Paihte tribe, and 8 species are only used by the Hmar tribe, as pictured in Venn’s diagram **(**Fig. 9**).**

### The market survey of WEVs

During the 24-month survey, 47 WEVs belonging to 38 genera and 27 families were found to be sold in the Bara bazaar (market) in Aizawl with a price range of 0.1–2.4 USD, depending upon the adequacy of the size. Based on the vendors’ attributes, most of these WEVs were gathered directly by the villagers from the forest and either directly sold by them or handed over to some commission agents. Based on these surveys, seasons play an important role in determining the price of the vegetables; some of the WEVs were sold throughout the year, including the species of *Lepionurus sylvestris*, *musa sp, Caryota urens, Caryota mitis Eurya acuminata, Calamus tenuis, Calamus erectus Marsdenia formosana, Marsdenia maculata* and *Ensete superbum*. Most products were organized on display as bundles or packets weighing ~ 20 g–1 kg. The *L. sylvestris* are sold in ~100g bundle at a price of 0.4–0.6 USD. *C. urens, C. mitis, C. erectus,* and *C. tenuis* are sold in piles of ~ 500 g at a great price of 1.2–2.4 USD, respectively*. Musa sp* are sold at 0.1USD per inflorescence. Interestingly*, E. acuminata,* leafy vegetables, are sold at 0.2–0.4 USD (~100g/Bundle), which is currently being used for the preparation of an important and popular dish of the Hmar tribe known as ‘Chartang’ and ‘Beipenek’. *M. formosana* and *M. maculata* are the two bitter leafy vegetables which have a great impact on promoting the cash income for the rural population in the study region, has been sold at 0.6–0.8 USD (~100g/ Bundle). These plants have always been highly valued by the majority of the ethnic group in the study area, currently in greater demand from the customer in the Bara bazar market in Aizawl. Moreover, based on the verbal interview with the local vendor informants, bamboo shoots can constitute important local commodities fetching high prices on local and regional markets and contribute to local cash income at the time of availability. The tender shoots of *D. hamiltonii* and* D. longispathus* are found to be sold during May-Oct with an excellent price range of 0.6–2.4 USD (~1kg/Bundle), respectively, where the price becomes higher at the time of fewness. Similarly, the tender shoot of *M. baccifera* offers a great price of 0.6–1.2 USD (~1 kg/ bundle), promoting income generation for the local people during April-Sept. These plants grow spontaneously and can be harvested from fallow land, home gardens, and agricultural land. The price fluctuates from season to season depending on supply chains' adequacy and availability. The parts sold, available season, and local market prices (USD) of WEVs sold in Bara Bazar are below **(**Table 6**).**

**Table 6:** List of common WEVs sold by vendors in the Bara Bazar market in Mizoram

Sl/no	Botanical name	Parts sold	Market price in USD	Available season
1	*Acmella oleraceae*	Leaves & shoot	0.6/Bundle(~250g)	Feb–Nov
2	*Aganope thyrsiflora*	Leaves &shoot	0.4–0.6/Bundle (~250g)	Feb–Apr
3	*Alocasia fornicata*	Flower bud	0.6–1.0/Bundle (~50g)	Jul–Sep
4	*Amaranthus viridis*	Tender shoot & leaves	0.4–0.6/ Bundle)	Apr–Sep
5	*Amomum dealbatum*	Inflorescence	0.6–0.7/ Pack (~200g)	Jan–Apr
6	*Amorphophallus napalensis*	Bulb	0.5–0.6/ Cup (~200ml)	Aug–Dec
7	*Aralia foliolosa*	Tender shoot & leaves	0.4–0.6 / Bundle(~100g)	Mar–Aug
8	*Bambusa tulda*	Tender shoot	0.6–2.4/ Bundle (~500g)	May–Oct
9	*Calamus erectus*	Tender shoot	1.2–2.4/ Pack (~ 500 g)	whole year
10	*Calamus tenuis*	Tender shoot	1.2–2.4/ Pack (~500g)	whole year
11	*Caryota mitis*	Tender shoot	1.2–2.4/ Pack (~ 500 g)	whole year
12	*Caryota urens*	Tender shoot	1.2–2.4/ Pack (~500g)	whole year
13	*Centella asiatica*	Whole plant	0.4–0.6/ Bundle (~20g)	Apr–Oct
14	*Chenopodium album*	Tender shoot & leaves	0.2–0.4/Bundle (~ 100 g)	Mar–May
15	*Clerodendrum bracteatum*	Tender leaves	0.6–0.7/ Bundle (~200g)	Jan–Oct
16	*Crotalaria tetragona*	Flower	0.4–0.6/ Pack(~50g)	Sep–Dec
17	*Dendrocalamus hamiltonii*	Tender shoot	0.6–2.4/ Bundle (~1 kg)	May–Oct
18	*Dendrocalamus longispathus*	Tender shoot	0.6–2.4/ Bundle(~1kg)	May–Oct
19	*Diplazium esculentum*	Leaves	0.2–0.6/ Bundle (~ 50 g)	Mar–Nov
20	*Dysoxylum excelsum*	Tender shoot & leaves	0.6–1.0/ Bundle(~50g)	Apr–Sep
21	*Ensete* *superbum*	Aerial pseudo stem	0.5/ Bundle (~ 500 g)	whole year
22	*Eryngium foetidum*	Leaves	0.2–0.4/ Bundle (~20g)	Mar–Nov
23	*Eurya acuminata*	Leaves	0.2–0.4/ Bundle (~ 100 g)	whole year
24	*Glinus oppositifolius*	Whole plant	0.4–0.6/ Bundle (~ 100 g)	Apr–Nov
25	*Gnetum gnemon*	Tender leaves & fruits	0.4–0.5/ Pack (~ 50 g)	Jun–Sep
26	*Houttuynia cordata*	Leaves	0.4–0.6/ Bundle (~ 100 g)	Feb–Aug
27	*Lepionurus sylvestris*	Leaves	0.4–0.6/ bundle (~50g)	Whole year
28	*Marsdenia formosana*	Leaves	0.6–0.8/ Bundle (~100g)	whole year
29	*Marsdenia maculata*	Shoot & leaves	0.6–0.8/ Bundle(~100g)	whole year
30	*Meloccana baccifera*	Tender shoot	0.6– 1.2/Bundle (~ 1 kg)	Apr–Sep
31	*Momordica charantia*	Fruits & leaves	0.6–1.2/ 1 kg	Apr–Oct
32	*Momordica* *dioica*	Fruits	0.7–1.4/ 1 kg	May –Jun
33	*Musa balbisiana*	Inflorescence	0.1/Inflorescence	whole year
34	*Musa paradisiaca*	Inflorescence	0.1/ Inflorescence	whole year
35	*Oroxylum indicum*	Fruits	0.2–0.4/ Pod	Aug–Nov
36	*Parkia timoriana*	Fruits	0.1–0.2/ Pod	Oct–Apr
37	*Picria fel-terrae*	Leaves	0.2–0.4/ Bundle(~50g)	May–Dec
38	*Rhynchotechum* *ellipticum*	Leaves	0.4–0.5/ Bundle (~ 100 g)	Apr–Aug
39	*Senegalia pennata*	Tender leaves & shoot	0.6/ Bundle(~30g)	Mar–May
40	*Senna occidentalis*	Leaves	0.4–0.6/ Bundle (~ 30 g)	May–Jul
41	*Solanum americanum*	Tender shoot & leaves	0.4–0.6/ Bundle (~ 250 g)	Feb–Oct
42	*Solanum torvum*	Fruits	0.4–0.6/ pack (~ 100 g)	May –Jul
43	*Solanum anguivi*	Fruits	0.6/ Pack (~100g)	Apr–Jul
44	*Solanum lycopersicum*	Fruits	0.6–1.2/1 kg	Feb–Dec
45	*Trevesia palmata*	Flower bud	0.6–1.2/ Pack (~250g)	Feb–April
46	*Wendlandia budleioides*	Flower bud	0.4–0.6/ bundle (~ 200 g)	Aug–Dec
47	*Zanthoxylum rhetsa*	Tender leaves & shoot	0.4–0.6/ Bundle (~20g)	Feb–April

### Novelty assessment

To assess the novelty of the encountered species, a comparative analysis was conducted with the previous Indian ethnobotanical studies (Table 1) and revealed 63 WEVs reported earlier as food items by different ethnic tribes. However, to the best of our knowledge, the following species such as *Picria-fel-terrae, Solena heterophylla, Musa paradisiaca, Aralia dasyphylla, Elatostema rupestre, Bambusa tulda,* and *Blumea myriocephala* have never been reported as edible vegetables and is reported for the first time in the present study.

## Discussion

WEVs served an important purpose as a daily food supplement among the two ethnic groups of Mizoram. The present ethnobotanical survey documented 70 species of WEVs belonging to 36 families from the two ethnic groups of Mizoram. A rich diversity of WEVs was documented from different ethnic groups in various geographical habitats. The number of species recorded in the present study was higher than 64 species reported from Soro District, Southern Ethiopia [43], Amuria District, Uganda (51 species)[44], Gansu–Ningxia–Inner Mongolia junction zone (53 species) [45] and lower than the reported species (90) from the Udhampur, J&K, India [39]. Fabaceae, Cucurbitaceae, Amaranthaceous, and Asteraceae represented the most diverse groups, possibly due to the sturdier adaptation potentiality of the species under these families over a wide range of environments. Similarly, this was reported in Mardin—Turkey—[46] and Jammu & Kashmir [39]. The present study revealed that the ethnic communities still depend on WEVs. The high usage of WEVs indicates a deep knowledge of ethnobotanical plants, easy availability, poor economic status of the local populace, and far-off residential places from the market [47, 48].

The life forms of WEVs in the present study reported that herbs were the most dominating species, supported by the findings of Li et al. [48]. On the contrary, Teklehaymanot and Giday [49] reported that trees are the most consumed growth form, followed by shrubs. This difference may come from the study area's ecological variance and vegetation types. Amente [50] further reported that shrubs exhibited Ethiopia's most dominant growth form of WEVs. The present study further indicated that leafy vegetables were the most highly consumed part by the inhabitants, supported by Singh et al. [51]

Due to their diverse edible parts and seasonality, different WEVs could be harvested in different seasons. Most of the WEVs in the present study were harvested during the rainy season, which was earlier reported as the peak season [52]. The local people know the exact time of the harvesting period, which could also help conservation. However, the method of harvesting in the present study area was rudimentary, thereby causing deleterious effects on the plant species. The methods involved cutting the whole tree rather than plucking off the edible portion to harvest WEVs such as *D. excelsum, W. bulloides, and X. rhetsa,*. This implies that proper management and harvesting patterns must be considered to minimize the detrimental effects [44]. Generally, WEVs provide an excellent source of nutrients and ecological security because of the disease’s resistance to growth in adverse climatic conditions and habitats [52]. This makes human diets more diverse during food shortages and is globally recognized as a key component in ecosystem-based adaptation and food scarcity [53]. This condition could inspire the locals to conserve WEVs resources and encourage domestication.

The JI for WEVs consumed between the two ethnic communities was relatively high (> 0.5), which reflects similarity in species viability. Although the two ethnic groups speak a distinct language, they have more or less similar choices in affinity towards WEVs consumption. This may be due to the intermixing of culture and tradition, mixed habitation, and the sharing of traditional knowledge among the two ethnic communities in the study area [4]. Apart from this, microclimatic conditions and variations in vegetation may influence the degree of similarity [54].

The study of ICF of food used categories revealed a high level of agreement among the informants in all used categories, indicating a more consistent use of WEVs among the two ethnic groups under study. In the present study, the food used categories were high for vegetables combined with dried fish, possibly due to the high number of UR for only one particular species consumed [55]. Similar results of high ICF values have also been reported from various parts of India [39, 51]. According to Mesfin et al. [56], ICF plays a significant role in plant species selection for further research concerning their chemical compositions against various ailments. Studies conducted in Pakistan also supported the present study's highest ICF value for Antiseptic [57].

Globally, indigenous communities utilize wild plants for food and to treat various diseases[20]. In the study area, decoction was ethnomedicine's most preferred mode of administration. Some traditional medicines have been reported to treat snakebites [58, 59]. The present study reported four WEVs used in treating snakebites: *M. balbisiana*, *A. viridis*, *E. superbum*, and *A. bunius*. The lowest ICF value recorded for snakebites in the present study was also comparable with the previous work [18]; however, it was found contrary where ICF value was highest for snakebites [60]. The low ICF values indicated that the informants have little agreement on using many species mentioned and documented [61]. For venomous snakebites, *A. bunius* mature leaves were normally used, which was also reported by Hazarika and Lalramchuana [62]. The whole plants of *A. viridis* were crushed, and the juice was taken out to treat snakebites in the present study, following the administration mode reported by Pakistan [63]. The juice of the tender shoot of *M.* *balbisiana* and *E. superbum* is applied to the bitten area by the Hmar people in the present study, which was similarly reported by Khiangte and Lalramnghinglova [64]. Several pharmacological investigations for snakebite venom properties have been carried out using traditional ethnomedicine [65, 66], and further investigation may lead to the discovery of active biomolecules with therapeutic potential. The present findings indicated that informants have good knowledge of WEVs, which was shared to a great extent among the local inhabitants. These WEVs are presently used among the major local populace as food and medicine. There was no standardized effective dosage of administrations on the use of medicinal plants.

Nevertheless, they were normally taken as food; for example, *S. americanum* was boiled for about 20 min and consumed to treat urinary problems. This study aligned with the literature reported from India [57]. In addition, *S.* *americanum* is an economically important plant due to its traditional use in the health care system to cure various ailments such as heart diseases, rheumatism, fever, hepatitis, and anti-tumour [67]. Various pharmacological studies revealed that *S. americanum* revealed various therapeutic potentials, including antioxidants, anti-inflammatory, anti-bacterial, and neuroprotective activities both *in vivo* and *in vitro* [7]. For dermatological problems, the raw plants of *S. anguivi* and *A. indica* were rubbed between the fingers and put onto the infected skin area.

Interestingly, some of these ethnomedicinal plants tended to treat more than one disease. For example, *S. anguivi* was used to treat three different ailments: dermatological problems, boils, and antiseptic. Likewise, *M. balbisiana* was employed to treat both diarrhoea and snakebites. In addition, many informants were found to use these medicinal plants orally, which agreed with the earlier studies [68, 69]. Due to the availability of micronutrients, bioactive compounds, and other pharmacological importance, WEVs have been considered a reliable and long-term solution for food security and discovering an alternative drug source [70]. Further investigations on species distribution are needed to address conservation concerns that may threaten such plants.

FL was evaluated for the most common ethnomedicinal plants cited by the informants based on their effectiveness in treating different diseases. *Picria-fel terrae* exhibited the highest FL values (100% and 95%) against hypertension and diabetes, respectively, similarly reported previously [18]. These results may further indicate that this plant species has a strong healing ability in the traditional medicinal practices of the region. Investigations on the bioactive compounds such as flavonoids, tannins, glycosides, saponin, and steroids, which might be responsible for its potential curing agents for diabetes, inflammation, diuretic, hepatoprotective, cancer, and asthma, have also been reported [71, 72]. High-fidelity medicinal plants showed the informants' preference for the given species to treat disease [60]. Therefore, a plant with high fidelity may be suggested for further investigation of the bioactive compounds for their high potential value [73]. *M. charantia* exhibited the second highest FL values (93%) against diabetes, which also agreed with the previous studies [74, 75]. In line with this, the potential application of *M. charantia* bioactive compounds in managing hyperglycemia and related chronic diseases was reported [76]. However, the low FL value of *S. pennata *and *E. foetidum *for treating food poisoning and malaria may be due to the rare occurrence of these diseases and, hence, the narrow distribution of information about their remedy in the study area. The plants having high FL values may be further investigated for their pharmaceutical and nutraceutical potential.

DMR implemented the status of plants to set conservation priority, whether they were under stress conditions or not, and the consequent factors that threatened the plants. Based on the relative benefits attained from each chosen plant species, it was conceivable to evaluate the relative importance and to check the major impact due to the over-exploitation of each plant species over other species in the study area [43]. In this study, *D. excelsum,* W. *budleioides,* and *R. ellipticum* are economically and domestically important plant species. As such species are harvested for timber, fuel wood, fodder, and medicinal purposes, they are over-exploited in addition to their food purpose. They may lead to rapidly declining populations in the study area. This was similarly described previously, in which the over-harvesting of multipurpose species was the main factor that threatened the plant species [77]. This demonstrated how the local people used the multipurpose plant species in various ways to meet their basic requirements, and are comparable to those of the study conducted on how they use the multipurpose plant species from Northwestern Ethiopia [78]. This clearly showed how the biotic pressure acts on the area's plant species, as supported by Panhwar et al. [79] and Razaq et al. [80]. In addition, habitat destruction, clearing of the virgin forest for rice cultivation, and collection of timber and fuelwood are the major problems leading to the disappearance of the natural products in the study area, similar to the case in the western region of Mizoram [81]. The local communities adopted the unsustainable and destructive harvesting technique of the study species, especially the tree species where the trees are damaged by the collector while collecting the tender leaves, fruits, and flowers for multipurpose use (e.g. *A indica, D. excelsum, and O. indicum*). This finding is consistent with the study [81] on ethnomedicinal plants of the western region of Mizoram. Such practices could severely affect plant growth and survival. The damage was more pronounced in trees. In shrub species, the damage was not that significant as it can be gathered from the ground. Digging the tubers and uprooting the whole plants were the most prevalent harvesting techniques for the herb species, consistent with a study of WEVs in the South Gondar Zone, Northwestern Ethiopia [78].

According to the IUCN Red List of Threatened Species (https://www.iucnredlist.org/), *E. superbum* is listed as near threatened in the present study, which needs prioritized settings for conservation through community awareness of affluence burden exerted on WEVs populations by the local inhabitants. One species is classified as data deficient; twenty-eight species are the least concerned, and forty-two species have not been evaluated. Moreover, the lack of appropriate data for prioritization conservation action critically hampers plant conservation efforts [82]. However, some of the taxa in the not evaluated category are cosmopolitan species, mainly constituted shrub and herb species, occurring abundantly in different ecological areas, which are not in danger of being lost.

In addition to the food value, the present study documented 47 species sold at the Mizoram local market (Bara Bazar). This finding was comparable to the market report on 42 species of WEVs in the Longding district of Arunachal Pradesh, India [83]. They further reported that similar WEVs sold, including *Musa sp., M. baccifera, D. hamiltoni, Z. rhetsa, C. bracteatum, E. foetidum, H. cordata* etc. in high price. Chaudhury et al. [84] reported 158 species of wild plants commonly sold from seven markets in the Eastern Himalayan state of Assam, India. In Mizoram, men are active in gathering vegetables; women are responsible for the selling of forest products in the local market in higher proportion than men, which was consistent with the study of the marketing potential of WEVs in Central India [85]. Bamboo and cane shoots were more in demand in the bara bazar market. They significantly impacted household income generation, as reported by Uprety et al. [86] in Nepal and Mishra et al. [85] in Central India. As per the quantity of the bundle size, *S. pennata, C. asiatica, and X. rhetsa* offer a great price in the under-studied market; this indicates traditional resource usage and the dependency of WEVs for promoting cash income. Some WEVs with good economic values were depleted due to over-exploitation and habitat destruction [86]. Most of the traditional WEVs reported by the ethnic communities could be propagated in the community land and the home garden by encouraging them to conserve, manage, and sustain their use to become more profitable cash crops [38, 87]. According to Łuczaj [88], the term 'herbophilia' could apply to the two ethnic tribes under-studied, in which the green parts of plants of numerous species are often used and highly prized.

The utilization of WEVs can make a positive impact in enhancing food security. It offers a rich variety of nutrition, for example, *Portulaca oleracea* in the present study contains a high omega 3 fatty acid, Vitamins, and minerals [6]*, *likewise, *Chenopodium album* is also rich in vitamins (A, C, K), iron and proteins [89]. Incorporating them into diets can contribute to overall health and well-being [22]. WEVs can thrive well in arid conditions, even when the conventional crop is affected by climate change, these resilient plants can serve as a buffer against food security. Their cultivation requires fewer resources, establishing sustainable agriculture. Moreover, WEVs in the present study offers a household cash economy, empowering local farmers by promoting domestication and commercialization that could contribute to the overall food security [90] and foster economic development in rural areas [91]. The local traditional knowledge is a treasure trove of wisdom passed down through generations and to keep this alive, documentation, for example, the present study that engaged the local communities in sharing their knowledge, cultural practices, and experience is essential. The community can share thoughts and use traditional plants sustainably by holding workshops, seminars, and knowledge-sharing sessions. Traditional knowledge can be included in curricula at educational institutions. To avoid over-exploitation, domestication should be promoted along with sustainable harvesting techniques. The preservation of traditional knowledge is an investment in our future well-being[31].

## Conclusion

This work highlighted the rich diversity of WEVs in Aizawl district of Mizoram and reported that Hmar and Paihte ethnic groups more or less share the same food habits. Informants have good knowledge of WEVs, which was shared with the inhabitants to a great extent. The major local populace is presently using these WEVs for food and medicine. Quantitative ethnobotanical survey of WEVs provides basic information for conservation, sustainable utilization of local WEVs, and preservation of local traditional knowledge of Mizoram.

Moreover, the study suggests investigating the pharmacological activity, particularly with traditional therapeutic plants, to validate their use—secondly, investigations on the nutritional content of all the recorded species. Introducing suitable modern techniques will also provide a substantial base for the commercial exploration of WEVs, which will be necessary for developing new food and use in the pharmaceutical industry.

## Data Availability

All the data used in this study are used in the manuscript.

## Abbreviations

WEVs: Wild edible vegetables
ICF: Informant’s consensus factor
FL: Fidelity level value
DMR: Direct matrix ranking
UR: Use reports
JI: Jaccard Index
MS Excel: Microsoft excel
IUCN: Union for conservation of nature
NE: Not evaluated
DD: Data deficient
NT: Nearly threatened
